# Relationship of urinary isoprostanes to prostate cancer occurence

**DOI:** 10.1007/s11010-012-1455-z

**Published:** 2012-09-16

**Authors:** Magdalena Brys, Agnieszka Morel, Ewa Forma, Anna Krzeslak, Jacek Wilkosz, Waldemar Rozanski, Beata Olas

**Affiliations:** 1 Department of Cytobiochemistry, Faculty of Biology and Environmental Protection, University of Lodz, Pomorska 141/143, 90-236 Lodz, Poland; 2 Department of General Biochemistry, Institute of Biochemistry, Faculty of Biology and Environmental Protection, University of Lodz, Pomorska 141/143, 90-236 Lodz, Poland; 3 2nd Department of Urology, Medical University of Lodz, Pabianicka 62, 93-513 Lodz, Poland

**Keywords:** Isoprostanes, Prostate cancer, Oxidative stress, Uric acid

## Abstract

To estimate the oxidative stress in patients with prostate cancer and in a control group, we used the biomarker of lipid peroxidation–isoprostanes (8-isoPGF_2_) and the level of selected antioxidants (glucose and uric acid [UA]). The level of urinary isoprostanes was determined in patients and controls using an immunoassay kit according to the manufacturer’s instruction. The levels of UA and glucose were also determined in serum by the use of UA Assay Kit and Glucose Assay Kit. We observed a statistically increased the level of isoprostanes in urine of patients with prostate cancer in compared with a control group. The concentration of tested antioxidants in blood from patients with prostate cancer was also higher than in healthy subjects. Moreover, our experiments indicate that the correlation between the increased amount of UA and the lipid peroxidation exists in prostate cancer patients (in all tested groups). Prostate cancer risk by urinary isoprostanes level was analyzed, and a positive association was found (relative risk for highest vs. lowest quartile of urinary isoprostanes = 1.6; 95 % confidence interval 1.2–2.4; *p* for trend = 0.03). We suggest that reactive oxygen species induce peroxidation of unsaturated fatty acid in patients with prostate cancer, and the level of isoprostanes may be used as a non-invasive marker for determination of oxidative stress. We also propose that UA may enhance the oxidative stress in patients with prostate cancer.

## Introduction

In different disorders, including cancer, oxidative stress is observed [1]. Oxidative stress leads to lipid peroxidation and may contribute to the pathogenesis of lesions in cancer, including prostate cancer [2], however, the mechanism of prostate carcinogenesis is not completely understood. In the presence of reactive oxygen species, double bonds of unsaturated fatty acids of phospholipids are oxidized. A scission of the oxidized polyunsaturated fatty acids results in the formation of phospholipid aldehydes such as oxidized phosphatidylcholine and aldehyde cleavage fragments including malonyldialdehyde, 4-hydroxynonenal, and acrolein [3]. Reactive oxygen species catalyzed peroxidation of arachidonic acid esterified in membrane phospholipids also leads to the formation of isoprostanes, which are prostaglandin-like compounds [4–9]. Oxidative stress may be determined by special biomarkers—the level of lipid hydroperoxides, conjugated dienes, thiobarbituric acid reactive substances, and reactive oxygen species.

The measurements of lipid peroxidation products in vitro relies on indirect methods. The specific and reliable markers of lipid peroxidation in vivo seem to be isoprostanes [5–7, 10–12], therefore, the aim of our study was to determine the level of urinary excretion of 8-isoprostaglandin F_2_ (8-isoPGF_2α_) in patients with prostate cancer and in healthy subjects, and also to estimate oxidative stress in patient groups and a control group on the basis of antioxidants (glucose and uric acid [UA]) in blood. However, recent studies suggest that UA can become a prooxidant [13, 14].

## Materials and methods

### Patients and samples

The blood and urine samples were collected from 304 patients who had been referred to the 2nd Clinic of Urology, Department of Urology, Medical University of Łódź, Poland, between February 2009 and January 2012.

Blood was collected into sodium citrate (5 mM final concentration). Blood (9 ml) was drawn between 8 a.m. and 8.30 a.m. and sampled from an antecubital vein. For each subject, we recorded blood platelet counts, red blood, white blood cell counts, and the level of selected antioxidants (in serum), as well as the medical history and medications used. A urine sample was also collected between 8 a.m. and 8.30 a.m. and immediately stored at −70 °C. The urine samples were collected from patients prior to prostate biopsy. The number of collection (for blood and urine) was one per patient. Demographic data and medical history were obtained at the entry of each patient to the study (Table 1). The final diagnosis of prostate cancer was based on histopathologic examination. Patients diagnosed with previous prostate tumors or with tumors located elsewhere were excluded. None of the recruited patients received preoperative chemo- or radiotherapy. A group of 233 healthy individuals were collected from the hospital from routine controls of health and used as control. They were non-related men, who have never been diagnosed with prostate tumors, other tumors, or chronic disease, and were randomly selected and frequency matched to the cases on age. The urine and blood samples were taken from patients or healthy subjects not taking any medications or addictive substances (including tobacco or alcohol), keeping a balanced diet (meat and vegetables), with similar socio-economic background, using no antioxidant supplementation. Cases were divided into three groups according to the histologic grade. Group I—gleason scores 2–4, group II—gleason scores 5–7, and group III—gleason scores 8–10. The volume of the prostate was calculated using the standard ellipsoid formula, width × height × length × *π*/6.

**Table 1:** Demographic and clinical characteristics of study subjects

	Prostate cancer (%)	Controls (%)
Sample size	*n* = 304	*n* = 233
Age, years	61.4 ± 9.26	65.2 ± 13.1
Serum PSA (ng/ml)^a^		
<4	15 (4.9)	155 (66.5)
≥4–10	36 (12.0)	78 (33.5)
≥10–100	215 (70.7)	0
>100	38 (12.4)	0
Gleason score		
2–4 (group I)	100 (33.0)	0
5–7 (group II)	155 (51.0)	0
8–10 (group III)	49 (16.0)	0
Stage		
T1-2N0M0	124 (41.0)	0
T3-4N0M0	82 (27.0)	0
TxN1 or M1	98 (32.0)	0
Prostate volume		
<30 ml	97 (32.0)	84 (36.0)
30 to 50 ml	124 (41.0)	91 (39.0)
≥50 ml	83 (27.0)	58 (25.0)

^a^ PSA (prostate specific antigen) as measured at the time of diagnosis

The protocol was passed by the Committee for Research on Human Subjects of the Medical University of Lodz No. RNN/59/09/KE.

### Measurement of PSA

The serum total PSA level was assayed by immunometric analysis using the Abbott IMx Total PSA assay kit (Abbott Laboratories).

### Measurement of lipid peroxidation

The level of 8-isoPGF_2α_ was estimated in urine samples from control subjects and from patients using non-specific immunoassay kit (Oxis International, Inc.) according to the manufacturer’s instructions.

### Measurement of uric acid and glucose levels

The levels of UA and glucose were determined in serum by the use of Uric Acid Assay Kit and Glucose Assay Kit (all Abcam, Cambridge, UK), respectively, according to the manufacturer’s protocol.

### Statistical analysis

The statistical analysis was done by several tests. All values in this study were expressed as mean ± SD. In order to eliminate uncertain data, the Q-Dixon test was performed. The statistically significant difference between the control group and patients was done by Mann–Whitney test. We used Cox regression analysis to assess the association of urinary isoprostanes with the risk of prostate cancer. Reported *p* values were two-sided. Probabilities were considered significant whenever *p* value was lower than 0.05. All analyzes were completed using STATA software (version 11.0 StataCorp., Texas, USA).

## Results

Our studies showed that the level of 8-isoPGF_2α_ in urine from patients with prostate cancer was markedly higher than the level of 8-isoPGF_2α_ in the control healthy subjects (Table 2). In control group, the level of 8-isoPGF_2α_ in urine was 259.4 ± 39.4 pg/mg of creatinine, whereas samples of patients with prostate cancer (gleason scores 2–4) contained 437.2 ± 112.4 pg of 8-isoPGF_2α_ /mg of creatinine. We also observed a statistically increased level of 8-isoPGF_2α_ in urine of patients with prostate cancer belonging to groups II and III in compared with a control group (Table 2). Moreover, the concentration of tested biomarker in urine from patients with more anaplastic prostate cancer (group III) was higher than in patients with prostate cancer classified as gleason scores 2–4 and 5–7 (about 20 %––group I vs. group II; and about 200 %––group III vs. group I) (Table 2).

**Table 2:** The level of 8-isoPGF_2α_ (the marker of lipid peroxidation) in urine and the level of selected antioxidants (glucose and UA) in serum from patients with prostate cancer, and in control urine and serum obtained from healthy volunteers

	Level of 8-isoPGF_2α_ (pg/mg creatinine)	Level of glucose (mg/dl)	Level of uric acid (mg/dl)
Control, *n* = 233 (A)	259.4 ± 39.4	74.9 ± 19.3	3.5 ± 1.2
Gleason score			
Group I, *n* = 100 (B)	437.2 ± 112.4	100.3 ± 14.9	5.6 ± 3.0
	(B vs. A, *p* < 0.001)	(B vs. A, *p* < 0.001)	(B vs. A, *p* < 0.01)
Group II, *n* = 155 (C)	742.3 ± 197.5	105.7 ± 29.7	8.2 ± 2.3
	(C vs. A, *p* < 0.001; C vs. B, *p* < 0.001)	(C vs. A, *p* < 0.001; C vs. B, *p* > 0.05)	(C vs. A, p < 0.001; C vs. B, *p* < 0.001)
Group III, *n* = 49 (D)	877.8 ± 217.4	110.6 ± 33.4	8.8 ± 2.5
	(D vs. A, *p* < 0.001; D vs. B, *p* < 0.001; D vs. C, *p* < 0.01)	(D vs. A, *p* < 0.001; D vs. B, *p* > 0.05; D vs. C, *p* > 0.05)	(D vs. A, *p* < 0.001; D vs. B, *p* < 0.001; D vs. C, *p* > 0.05)
Stage classification			
T1-2N0M0, *n* = 124 (E)	570.4 ± 82.5	99.4 ± 14.2	6.6 ± 2.7
	(E vs. E, *p* < 0.001)	(E vs. E, *p* < 0.001)	(E vs. E, *p* < 0.01)
T3-4N0M0, *n* = 82 (F)	927.1 ± 281.4	111.3 ± 30.4	9.0 ± 2.5
	(F vs. A, *p* < 0.001; F vs. E, *p* < 0.001)	(F vs. A, *p* < 0.001; F vs. E, *p* < 0.05)	(F vs. A, *p* < 0.001; F vs. E, *p* < 0.001)
TxN1 or M1, *n* = 98 (G)	935.3 ± 254.6	113.7 ± 29.5	9.2 ± 2.4
	(G vs. A, *p* < 0.001; G vs. E, *p* < 0.001; G vs. F, *p* < 0.05)	(G vs. A, *p* < 0.001; G vs. E, *p* > 0.05; G vs. F, *p* > 0.05)	(G vs. A, *p* < 0.001; G vs. E, *p* < 0.001; G vs. F, *p* > 0.05)

Results are mean ± SD. The statistical analysis was done by Mann–Whitney test

We observed higher level of UA and glucose in serum of all prostate cancer patients (groups I, II, and III) than in control group (Table 2).

In this study, we did not find statistically significant correlation between concentrations of investigated compounds (8-isoPGF_2α,_ UA, and glucose) and patient’s age (data not shown). The correlation between the increased amount of UA and changes in the level of 8-isoPGF_2α_, in patients with prostate cancer (classified as groups I, II, and III [gleason score] and for stage classification) is presented in Table 3.

**Table 3:** The correlation between the selected parameters of oxidative stress—the level of 8-isoPGF_2α_ and the level of UA (for classifications—gleason score and stage classification)

	The correlation between the level of isoprostanes (pg/mg creatinine) and the level of uric acid (mg/dl)
Gleason classification:	
Group I	*r* = 0.7781 (*p* < 0.001)
Group II	*r* = 0.7653 (*p* < 0.001)
Group III	*r* = 0.6194 (*p* < 0.001)
	The correlation between the level of isoprostanes (pg/mg creatinine) and the level of uric acid (mg/dl)
Stage classification:	
T1-2N0M0	*r* = 0.7234 (*p* < 0.001)
T3-4N0M0	*r* = 0.7008 (*p* < 0.001)
TxN1 or M1	*r* = 0.6056 (*p* < 0.001)

Table 4 shows relative risk of prostate cancer by quartile of serum isoprostanes. These analyzes were run with adjustment for age. We observed a significant positive trend in these data.

**Table 4:** Relative risk of prostate cancer by quartile of urinary isoprostanes

Quartile of isoprostanes (pg/mg creatinine)	No. of cases (%)	Relative risk^a^	*P* for trend^b^
		(95 % CI)	
≤342.3	49 (16.1)	1.00 (reference)	
342.4–684.5	87 (28.6)	1.2 (0.7–1.6)	
684.6–1,026.8	135 (44.4)	1.4 (0.80–2.2)	
≥1,026.9	33 (10.9)	1.6 (1.2–2.4)	0.03

^a^ Adjusted across two age categories: 40–59, ≥60 years

^b^ Cochran–Armitage test for trend

*CI* confidence interval

## Discussion

Oxidation of polyunsaturated fatty acids changes the biological activity of phospholipids that is important for the integrity of cellular membranes. Two pathways of lipid peroxidation can occur: enzymatic and nonenzymatic. Free polyunsaturated fatty acids can be oxidized by multiple enzymes forming the reactive lipid mediators such as prostaglandins, thromboxanes, prostacyclins, lipoxins, and hepoxylins. Nonenzymatic lipid peroxidation process initiated by reactive oxygen species leads to the formation of different products, mainly isoprostanes, isothromboxanes, isolevuglandins, and isofuranes. Isoprostanes are the most important biomarkers of oxidative stress in human diseases [15–18]. In our study, isoprostanes were estimated in urine of prostate cancer patients and healthy volunteers. Measurement of 8-isoPGF_2α_ in a single sample of urine represents daily secretion of isoprostanes, which are the end products of peroxidation of arachidonic acid. The source of 8-isoPGF_2α_ may be oxidized arachidonic acid derived from different cells including neurons, blood platelets, and erythrocytes [5–7, 10–12].

This study, for the first time, provides evidence that patients with prostate cancer have extremely high level of urinary 8-isoPGF_2α_ (measured by using a sensitive immunoassay). It indicates that the oxidative stress takes place. The obtained results are consistent with those in the literature [2]. Barocas et al. [2] measured (by gas chromatography/negative ion chemical ionization mass spectrometry) the urinary level of F_2_-isoprostane, and they observed a statistically significant trend in prostate cancer risk with increasing of F_2_-isoprostane level. They observed that F_2_-isoprostane level was higher not only in patients with prostate cancer, but also in patients with high-grade prostatic intraepithelial neoplasia.

The presence of various antioxidants (UA, glutathione, and glucose) in blood is responsible for the protection against the oxidative stress [19]. The most potent of them is UA. This final metabolite of purine catabolism is a biomarker of oxidative stress, but it can also act as a prooxidant [13, 14]. The high level of glucose may also play a role of the inducer of oxidative stress [19]. In our study, the level of UA and glucose in serum was higher in patients with prostate cancer than in control. However, the levels of serum glucose among all tested groups of patients (for Gleason classification and stage classification) were the same. This study provides more information about mechanisms of oxidative stress in prostate cancer. The first time, our results showed that the elevated level of UA may play an important role in the oxidative stress in prostate cancer patients. Experiments presented here showed that there was a correlation between the increased amount of UA and the oxidative stress in patients with prostate cancer. It should be underlined that in our experiments, samples from patients were taken before surgery, and prostate cancer patients have not had preadjuvant therapy.

Our results support the hypothesis that oxidative stress (measured by various biomarkers, including lipid peroxidation) may play a role in prostate cancer. We also presented that in prostate cancer patients, the level of oxidative stress was not dependent on patient’s age. However, further studies are needed to evaluate the impact of pharmacologic treatment on modification of lipids in prostate cancer. The next step of future studies is to evaluate the role of various compounds (antioxidants and prooxidants) in the oxidative stress in patients with prostate cancer.
